# Regulation of Ammonium Cellular Levels is An Important Adaptive Trait for the Euhalophytic Behavior of *Salicornia europaea*

**DOI:** 10.3390/plants9020257

**Published:** 2020-02-17

**Authors:** Jinbiao Ma, Valerio Cirillo, Dayong Zhang, Albino Maggio, Lei Wang, Xinlong Xiao, Yinan Yao

**Affiliations:** 1 CAS Key Laboratory of Biogeography and Bioresources in Arid Land, Xinjiang Institute of Ecology and Geography, Urumqi 830011, China; majinbiao@ms.xjb.ac.cn; 2 Department of Agricultural Sciences, University of Naples Federico II, Via Università 100, 80055 Portici, Italy; valerio.cirillo@unina.it (V.C.); almaggio@unina.it (A.M.); 3 State Key Laboratory of Crop Genetics and Germplasm Enhancement, Cotton Hybrid R & D Engineering Center (the Ministry of Education), College of Agriculture, Nanjing Agricultural University, Nanjing 210095, China; cotton.z@126.com; 4 State Key Laboratory of Desert and Oasis Ecology, Xinjiang Institute of Ecology and Geography, Chinese Academy of Sciences, Urumqi 830011, China; egiwang@ms.xjb.ac.cn; 5 College of Life Sciences and Engineering, Southwest University of Sciences and Technology, Mianyang 62101, China

**Keywords:** salinity, NH_4_^+^ assimilation enzymes, NH_4_^+^ detoxification, salt stress, glycophytes, halophytes

## Abstract

Salinization of agricultural land is a devastating phenomenon which will affect future food security. Understanding how plants survive and thrive in response to salinity is therefore critical to potentiate tolerance traits in crop species. The halophyte *Salicornia europaea* has been used as model system for this purpose. High salinity causes NH_4_^+^ accumulation in plant tissues and consequent toxicity symptoms that may further exacerbate those caused by NaCl. In this experiment we exposed Salicornia plants to five concentrations of NaCl (0, 1, 10, 50 and 200 mM) in combination with two concentrations of NH_4_Cl (1 and 50 mM). We confirmed the euhalophytic behavior of Salicornia that grew better at 200 vs. 0 mM NaCl in terms of both fresh (+34%) and dry (+46%) weights. Addition of 50 mM NH_4_Cl to the growth medium caused a general growth reduction, which was likely caused by NH_4_^+^ accumulation and toxicity in roots and shoots. When plants were exposed to high NH_4_Cl, high salinity reduced roots NH_4_^+^ concentration (−50%) compared to 0 mM NaCl. This correlates with the activation of the NH_4_^+^ assimilation enzymes, glutamine synthetase and glutamate dehydrogenase, and the growth inhibition was partially recovered. We argue that NH_4_^+^ detoxification is an important trait under high salinity that may differentiate halophytes from glycophytes and we present a possible model for NH_4_^+^ detoxification in response to salinity.

## 1. Introduction

Salt stress is one of the most detrimental abiotic stresses perturbing plants ability to grow. It is estimated that salinization affects 20% of arable soils and is further expanding [1]. Much effort has been dedicated to improve both crop management in saline environments and crop tolerance to high salinity [2]. With respect to plants, halophytes have profitably been used as model systems to unravel key mechanisms and tolerance traits that differentiate them from glycophytes [3,4,5,6], with the ultimate goal of transferring and/or potentiating those mechanisms in crop plants [7,8]. Among halophytic species, euhalophytes can stand and grow well in up to 500 mM NaCl [5] in nature, a concentration that would be fatal to the majority of glycophytes, including most crops [9].

*Salicornia europaea* L. is one of the most studied euhalophytes, growing in coastal areas and inland salt marshes. It is a salt-accumulating species from the *Chenopodiaceae* family and the main adaptive traits allowing this specie to thrive in salty environments are well known. Among them, ion compartmentalization, regulation of intracellular osmotic balance and cellular turgor control are considered key determinants of its halophytic behavior [9,10,11,12,13,14].

Ion uptake and translocation within the plant is affected upon exposure to high concentrations of NaCl, with differences between halophytes and glycophytes [15]. In glycophytes, salt stress causes NH_4_^+^ accumulation in plant cells, which is toxic [16] and may further exacerbate damages due to NaCl [17,18]. Moreover, the level of free NH_4_^+^ content in plants has been negatively correlated with salt stress tolerance [19,20,21]. The ammonium content in rice increased three to four times when seedlings were treated with 100 mM NaCl. Salt stress induced NH_4_^+^ accumulation has been associated to the inactivation of specific enzymes involved in NH_4_^+^ homeostasis [3,18], including glutamine synthetase (GS) and glutamate dehydrogenase (GDH) which can maintain NH_4_^+^ cellular concentrations below toxicity levels by incorporating it into amino acids [22,23]. It has also been reported that NH_4_^+^ fertilization in both *Populus simonii* and *Sesamum indicum* worsens the detrimental effects of salt stress on physiological and growth parameters, which indicates that high NH_4_^+^ under salinity does not help plants cope with salinity [24,25]. Despite this, NH_4_^+^ nutrition has been reported to enhance salt tolerance in citrus plants [26], *Spartina alterniflora* [27] and other wetland plants [28]. In these experiments, since plants grown with NH_4_^+^ were healthier irrespectively of salt stress, it was concluded that these species preferred NH_4_^+^ as a form of nitrogen form rather than NO_3_^−^ [29]. In contrast to glycophytes, it has recently been shown in the halophytic *Salicornia europaea* that several genes involved in NH_4_^+^ assimilation and translocation were upregulated when NaCl was added to the growth medium, indicating that the control of NH_4_^+^ levels in the presence of high concentrations of NaCl may have physiological relevance for this species [30].

Based on these considerations, we hypothesized that the ability of Salicornia to maintain NH_4_^+^ levels below toxicity limits is important for this species to cope with high salinity and may be a distinctive trait for halophytes as compared to glycophytes. Several reports have indeed documented higher expression of GS and GDH in halophytes as compared to glycophytes [31,32,33]. Here we demonstrate that high GS and GDH activity prevented tissue NH_4_^+^ accumulation in Salicornia plants grown at 200 mM NaCl. However, when extra NH_4_^+^ was exogenously supplied, the euhalophytic Salicornia lost its ability to thrive in a salty environment and behaved as a regular halophyte. These results bring upfront, for the first time, the mechanism of NH_4_^+^ detoxification as key determinant of plant growth under high salinity.

## 2. Results

### 2.1. Effects of NH_4_Cl/NaCl on Growth and Morpho-Physiological Traits

**Figure 1:**
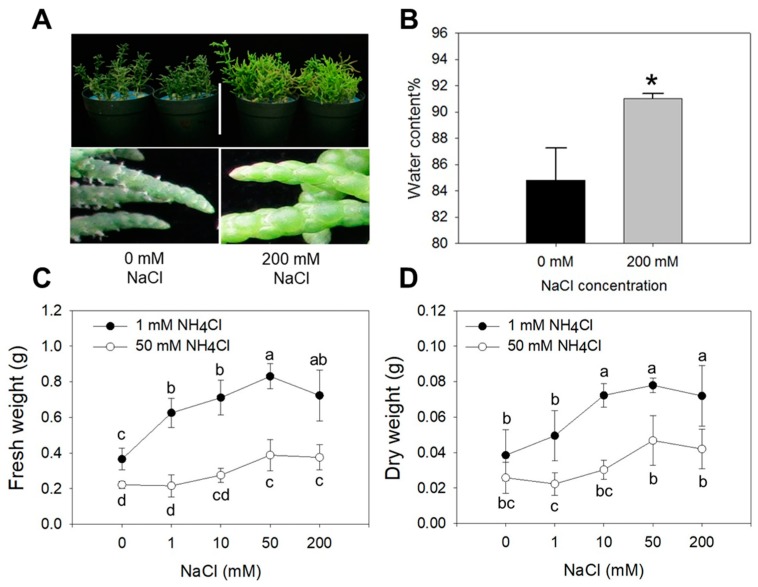
*S. europaea* water status and biomass accumulation under different NaCl/NH_4_Cl treatments. (**A**) Photos of *S. europaea* grown in NaCl-free nutrient solution or with addition of 200 mM NaCl. (**B**) Shoots water content of *S. europaea* under NaCl-free or 200 mM NaCl treatments. Asterisk represents significant differences compared to the control (0 mM NaCl) according to t-test (*p* < 0.05). (**C**) Fresh weight and (**D**) dry weight of *S. europaea* grown on agar medium at increasing NaCl levels and two NH_4_Cl levels. Means with the same letter are not significantly different according to the Duncan’s test (*p* < 0.05). Error bars represent the standard error.

Plants hydration state (Figure 1A) and water content (Figure 1B) were both higher at 200 mM NaCl compared to 0 mM NaCl. In the presence of 1 mM NH_4_^+^, whole plant fresh weight and dry weight significantly increased upon addition of NaCl to the growth medium (Figure 1C,D). At higher NH_4_Cl (50 mM), plants manifested symptoms of ammonium stress and both whole plant fresh and dry weights were reduced compared to 1 mM NH_4_Cl treatment at all NaCl concentrations tested (Figure 1C,D).

**Figure 2:**
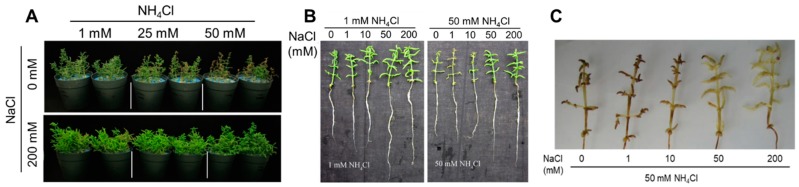
(**A**) *S. europaea* phenotype as affected by two concentrations of NaCl in combination with three concentrations of NH_4_Cl. (**B**) Whole seedlings of *S. europaea* under NaCl/NH_4_Cl treatments. (**C**) Detection of peroxides using DAB staining on *S. europaea* under 50 mM NH_4_Cl and increasing concentrations of NaCl.

Remarkably, under low NaCl and high NH_4_Cl, the conditions which showed the smallest NH_4_Cl effect (see differences at 0 mM NaCl vs. higher concentrations), the plant stem apex was yellow and wilted (Figure 2A,B). DAB staining also revealed symptoms of oxidative stress in shoot tips and apex tissues (Figure 2C).

Under higher NaCl treatment (50–200 mM), the ammonium toxicity phenotype was partially relieved, although the extra ammonium seemed to suppress the euhalophytic behavior of Salicornia (Figure 1C,D). It is worth noting that lower biomass accumulation and smallest differences in terms of plant fresh weight and dry weight with respect to low/high NH_4_Cl were both found at 0 mM NaCl treatment. These results confirmed that Salicornia plants need NaCl to grow well, but also indicate that high NH_4_ concentrations in the growth medium may become a more serious problem when plants have to deal with it under optimal growth conditions (high NaCl) (Figure 1A,B).

### 2.2. Shoot and Root Ionic Profile in Response to Increasing NaCl and NH_4_Cl Concentrations

**Figure 3:**
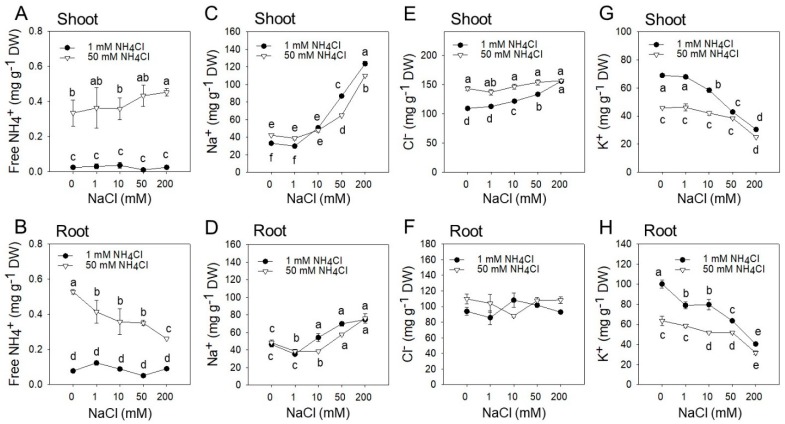
Ion content of shoots (top panel, **A**-**C**-**E**-**G**) and roots (bottom panel, **B**-**D**-**F**-**H**) of *S. europaea* in response to five NaCl concentrations (0, 1, 10, 50, 200 mM) in combination with two NH_4_Cl concentrations (1 and 50 mM). Means with the same letter are not significantly different according to the Duncan’s test (*p* < 0.05). Error bars represent the standard error.

At 1 mM NH_4_Cl, the NH_4_^+^ content in shoots and roots did not change at increasing salinity (Figure 3A,B). The tissue NH_4_^+^ content significantly increased in roots and shoots at 50 mM NH_4_Cl (approximately 5-fold increase at 0 mM NaCl). However, the NH_4_^+^ content in roots gradually decreased at increasing NaCl (50% less from 0 to 200 mM NaCl). In contrast, the NH_4_^+^ concentration moderately increased in shoots from 0 to 200 mM NaCl (Figure 3A,B). At higher NaCl concentrations, Na^+^ in shoot tissues rapidly increased up to 3-fold from 0 to 200 mM NaCl, while only 36% increase in roots. Addition of 50 mM NH_4_^+^ partially counteracted the Na^+^ accumulation in shoots at 50 and 200 mM NaCl; whereas the same effect was observed in roots at 10 and 50 mM NaCl (Figure 3C,D). On the other hand, the shoot Cl^−^ content increased at moderate rate from 0 to 200 mM NaCl, reaching 155.7 mg/g dry weight at 200 mM vs. 109.2 mg/g dry weight at 0 mM 50 mM NH_4_Cl treatment caused 16.5% increase of the shoot Cl^−^ concentration compared to plants at 1 mM NH_4_Cl (average of all NaCl levels) but nearly constant at increasing NaCl concentrations (Figure 3E). In roots, the Cl^−^ concentration was similar for the two NH_4_Cl levels and at increasing salinity, indicating that most Cl^−^ is translocated to the shoots (Figure 3F). The K^+^ content gradually decreased in both shoots and roots at increasing NaCl, likely due to competition effects with Na^+^ and, at higher NH_4_Cl, likely due to competition effects with NH_4_^+^ (Figure 3G,H). The reduced K^+^ in the presence of NH_4_^+^ could have caused some turgor loss, which we observed under the 0 mM NaCl/50 mM NH_4_Cl combination (Figure 2A,B).

### 2.3. Expression Pattern of Genes Involved in the Ammonium Assimilation Metabolism in Response to Increasing NaCl and NH_4_Cl Concentrations

**Figure 4:**
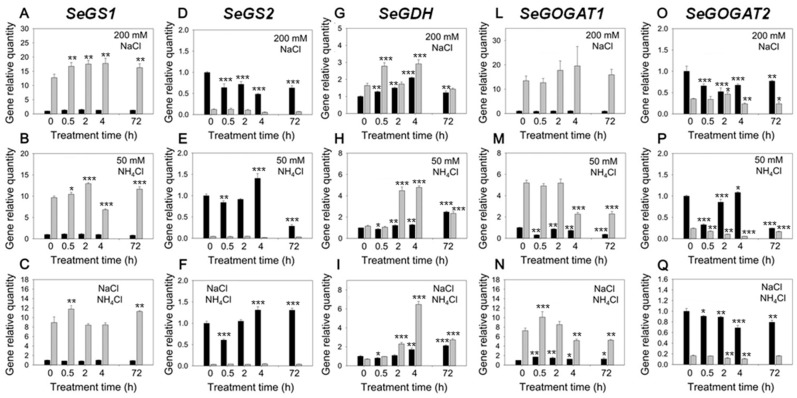
GS, GDH and GOGAT gene expressions in shoot (black bars) and root (grey bars) tissues of Salicornia plantlets treated with 200 mM NaCl (top panel—**A**, **D**, **G**, **L**, **O**), 50 mM NH_4_Cl (middle panel—**B**, **E**, **H**, **M**, **P**) and 200 mM NaCl + 50 mM NH_4_Cl (bottom panel—**C**, **F**, **I**, **N**, **Q**) for 0 h, 0.5 h, 2 h, 4 h, 72 h. Error bars represent the standard deviation. Asterisks (* = *p* < 0.05; ** = *p* < 0.01; *** = *p* < 0.001) indicate significant differences between each time point and the 0h point for shoots (black bars) and roots (grey bars) according to ANOVA and LSD post-hoc analysis (*n* = 3 biological replicates).

To further understand the molecular mechanisms that could mediate and explain Salicornia responses to NH_4_Cl/NaCl interactions, the expression of genes related to ammonium assimilation were detected using qRT-PCR. *GS1,* glutamine synthetase, (unigene5393_All) was expressed only in roots and showed a continuous increase from 0–4 h, with high expression at 72 h, under 200 mM NaCl (Figure 4A–C). However, *GS2* (unigene29790_All) was mainly expressed in shoot tissues and had a slight decrease under 200 mM NaCl treatment. A 50 mM NH_4_Cl treatment caused a short-term induction of this gene, which was inhibited after longer time (72 h). Interestingly, this inhibition was removed under 200 mM NaCl (Figure 4D–F). *GDH,* glutamate dehydrogenase (unigene5295_All) was expressed in both roots and shoots, and it was induced by 200 mM NaCl. *GDH* expression in roots was significantly upregulated by NH_4_Cl with up to four-fold increase after 2 h. The gene expression decreased at 72 h, but the transcript abundance was still higher than the 0 h control. The induction in shoots was delayed compared to roots, but the expression level significantly increased after 72 h. *GDH* expression in roots under both 50 mM NH_4_Cl and 200 mM NaCl increased up to seven- fold after 4h, it then decreased at 72 h but still had three-fold induction compared to control plants. The induction in shoots was delayed and the expression level gradually increased after 72 h (Figure 4G–I). *GOGAT1* (glutamate synthase) (unigene54171_All) was mainly expressed in roots and its expression was not significantly altered by 200 mM NaCl, yet it was inhibited by sole 50 mM NH_4_Cl treatment (Figure 4L–N). The same pattern was found for *GOGAT2* in the 50 mM NH_4_Cl treatment both in shoots and roots. However, the relative expression of this gene was also down-regulated at 200 mM NaCl (Figure 4O–Q). Under the combined 50 mM NH_4_Cl/200 mM NaCl treatment, GOGAT was inhibited also (Figure 4L–Q).

### 2.4. Effects of NH_4_^+^/Na^+^ on Enzyme Activities Related with Ammonium Assimilation Metabolism

**Figure 5:**
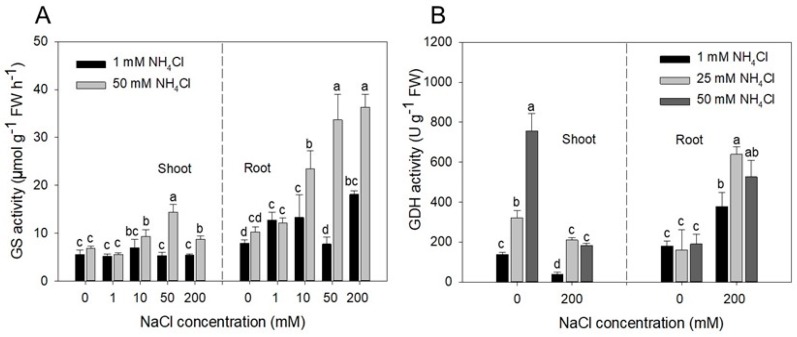
Activity detection of ammonium assimilation related enzymes under NaCl/NH_4_Cl treatments. (**A**) Glutamine synthase (GS) activity. (**B**) Glutamate dehydrogenase (GDH) activity in shoot and root tissues of Salicornia plantlets. Means with the same letter are not significantly different according to the Duncan’s test (*p* < 0.05). Error bars represent the standard error.

The glutamine synthetase (GS) activity was induced by 50 mM NH_4_Cl especially in roots and at higher NaCl concentrations (50 and 200 mM) (Figure 5A). Root GS activity was also moderately induced by NaCl (see GS response pattern at 1 mM NH_4_Cl). In the absence of NaCl, the glutamate dehydrogenase (GDH) activity was induced by NH_4_^+^ only in the shoots (Figure 5B), with a remarkable effect of the 50 mM treatment, which increased it by 4-fold compared to 1 mM NH_4_Cl. The presence of 200 mM NaCl in the growth medium significantly inhibited the GDH enzymatic activity in the shoots, while enhanced it in the roots (Figure 5B). Addition of 25 or 50 mM NH_4_Cl partially counteracted the NaCl induced GDH inhibition in the shoots, whereas it further enhanced it in the roots (Figure 5B).

## 3. Discussion

### 3.1. Regulation of NH_4_^+^ Toxicity is a Key Determinant of *Salicornia* Growth at High Salinity

Salicornia is one of the most known salt-accumulating halophytes and has been studied for its potential uses in agriculture [34] but also to unravel fundamental mechanisms underlying salt stress tolerance [35]. Most work with halophytes, including Salicornia, has focused on ion compartmentalization, regulation of intracellular osmotic balance and the control of cellular turgor as key determinants of their exceptional tolerance to high salinity [9,13,30]. More recently, large-scale de novo transcriptome analysis of gene expression in salinized roots of the euhalophyte *Salicornia europaea* [30] has also revealed that key genes involved in NH_4_^+^ assimilation are upregulated in response to salinity, a response which we further investigated. We confirmed the euhalophytic response of Salicornia [34] with an improved growth and Relative Water Content at 200 mM NaCl (Figure 1). Addition of NH_4_Cl to the growth medium significantly reduced plant growth, with largest effects at higher salinity (Figure 1). Despite the relatively moderate impact on plant growth in the absence of NaCl, NH_4_Cl caused tissue browning and some tip burns (Figure 2). This was most likely due to a general metabolic and growth impairment caused by the lack of Na^+^, which is the main osmoregulator in Salicornia and most halophytes [9,33], rather than specific NH_4_^+^ or Cl^−^ toxicity, since these symptoms were not observed at higher NaCl and/or NH_4_Cl treatments. Under these conditions, the presence of K^+^ could have at least partially replaced the function of Na^+^. Nevertheless, the K^+^ level was significantly reduced in both shoots and roots by 50 mM NH_4_Cl at low salinity (Figure 3G,H), probably due to competition effects of K^+^ with NH_4_^+^. This possibility is consistent with a reduced plant hydration state we observed in plants growing at low NaCl/high NH_4_Cl (Figure 1), a condition that may eventually lead to tissue desiccation if an excess of NH_4_^+^ in the medium persists [36].

With respect to other ions, the accumulation of Na^+^ and Cl^−^ in roots and shoots was consistent with most published literature [37,38]. Na^+^ increased in both shoot and roots at increasing salinity (Figure 3C,D) and it was moderately reduced by NH_4_Cl, possibly due to competition effects with NH_4_^+^ [39]. The concentration of Cl^−^ was relatively stable and was not affected by NH_4_Cl in the roots, whereas it moderately increased with salinity in the shoots, a response already observed for Salicornia and other halophytes [40]. The higher shoot Cl^−^ concentration in 50 mM NH_4_Cl treated plants also demonstrates that the growth inhibition observed in response to NH_4_Cl cannot be attributed to the effect of Cl^−^ per se since the largest growth differences were observed at 200 mM NaCl, where the shoot Cl^−^ was similar in 1 mM and 50 mM NH_4_Cl treated plants (Figure 3E,F). In contrast, the NH_4_^+^ accumulation pattern in roots and shoots was quite remarkable and, to our knowledge, it has never been documented before in response to salinity in Salicornia (Figure 3A,B). At 50 mM NH_4_Cl, the NH_4_^+^ concentration increased in both shoots (27-fold vs. 1 mM NH_4_Cl) and roots (11-fold vs. 1 mM NH_4_Cl). Most interestingly, NH_4_^+^ slightly increased in shoot upon salinization, whereas it significantly decreased in the roots. All together the ion accumulation patterns attributes mostly to NH_4_^+^ toxicity the observed growth inhibition. However, the correspondence between the root NH_4_^+^ decline (Figure 3B) and partial growth recovery (Figure 1C,D) at high NaCl also indicates that high salinity may activate specific NH_4_^+^ detoxification responses in Salicornia. Most toxic effects of NH_4_^+^ have been attributed to ionic imbalance and intercellular pH disturbance, and other mechanisms of action which are not fully understood [41,42,43]. NH_4_^+^ toxicity is enhanced by salinity in glycophytes [18], due to the inactivation by NaCl of NH_4_^+^ assimilating enzymes, and consequent accumulation of free NH_4_^+^ in plant tissues [44,45]. Plants ability to preserve the activity of NH_4_^+^ assimilation enzymes can therefore be important to regulate cellular NH_4_^+^ levels under salt stress. Consistent with this hypothesis, our data show that, in contrast to glycophytes [46], the euhalophyte *S. europaea* does not accumulate much NH_4_^+^ in the shoot and it remarkably reduces it in the roots when exposed to increasing salinity (Figure 3A,B).

### 3.2. NH_4_^+^ Detoxification is Mediated by Salt Induced Activation of the NH_4_^+^ Assimilation System

Salicornia plants grown under salinity have a higher NH_4_^+^ assimilation activity compared to glycophytes [31,33]. We found that the relative expression of the genes encoding for GS and GDH was significantly upregulated in plants grown at 200 NaCl compared to non-salinized control plants (Figure 4). These results support the hypothesis that in saline environment, Salicornia may control NaCl-induced NH_4_^+^ accumulation through activation of the GS/GDH mediated NH_4_^+^ detoxification system. This control occurs in roots via GS and GDH upregulation and in shoots mostly via GDH upregulation (Figure 4A–D–G). To better understand the role of NH_4_^+^ assimilation enzymes in response to salinity, we artificially altered the steady-state control of NH_4_^+^ levels under high salt by adding an excess of NH_4_^+^ to the growth medium. Similar to the NaCl treatment, addition of 50 mM NH_4_Cl caused an upregulation of NH_4_^+^ assimilation genes, which was maintained under the 200 mM NaCl/50 mM NH_4_Cl treatment (Figure 4 A–I). However, root and shoot accumulation of NH_4_^+^ at 50 mM NH_4_Cl also indicated that GS and GDH activities are insufficient to incorporate all available NH_4_^+^ into aminoacids and to maintain cellular NH_4_^+^ below toxicity levels (Figure 3A). These results were confirmed by the GS and GDH enzymatic activites which were both higher in roots at 200 mM NaCl (Figure 5). High NH_4_Cl and NaCl levels both enhanced glutamine synthetase (GS) activity in roots (Figure 5A), as already reported [47]. GDH activity increased in roots exposed to 200 mM NaCl whereas it was reduced in shoots. Interestingly, root GDH activity was severely inhibited by the overall hypo-osmotic stress of the plants (lack of Na^+^). In salt-free environment, GDH activity did not change upon exposure to high NH_4_^+^ levels (Figure 5B), leading to root over-accumulation of NH_4_^+^ compared to shoots (Figure 3A,B). Consistent with the lower NH_4_^+^ concentrations detected in the shoots, in the absence of NaCl, GDH activity was high in the shoot (Figure 5B). When the external concentration of NaCl was within optimal ranges for Salicornia, root GDH activity was restored and NH_4_^+^ was rapidly incorporated into aminoacids at root level, thus reducing the translocation of the ion to the shoot [48]. GS and GDH activities are generally unaffected or even inhibited by salt stress in most glycophytes [3,18,31,33]. An exceptionally efficient NH_4_^+^ detoxification ability of Salicornia, and possibly other euhalophytes could represent an important trait determining its/their halophytic behavior. It has been recently reported [43] that the NH_4_^+^ assimilation pathway, especially with respect to reduced GS and GOGAT enzyme activities, is weakened under high NH_4_^+^ stress in Arabidopsis plants, which exhibited an NH_4_^+^-sensitive phenotype typical of glycophytes.

### 3.3. Role of GOGAT in NH_4_^+^ Detoxification and Osmoprotection

**Figure 6:**
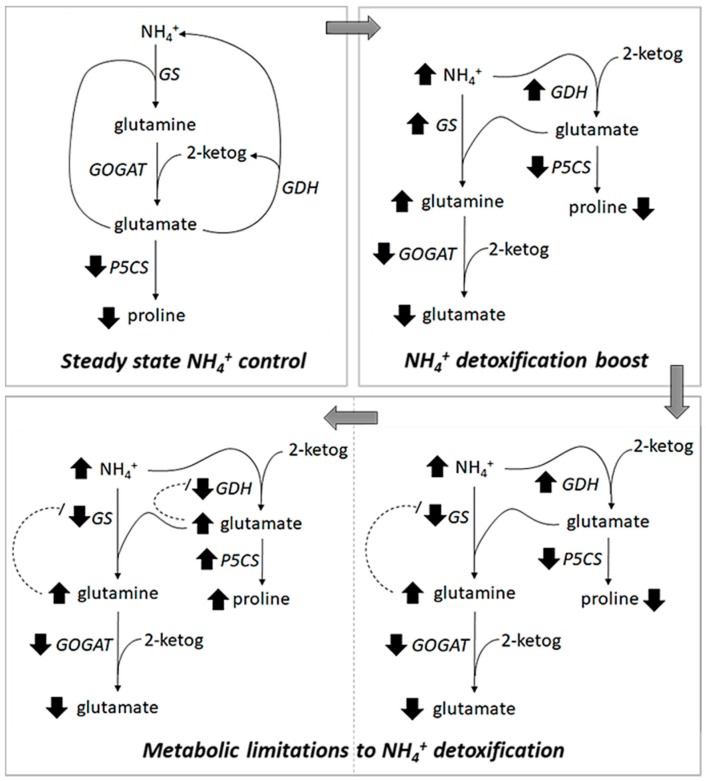
*A proposed model for NH_4_^+^ detoxification in plants.* A model for ammonium assimilation in response to direct/indirect causes of NH_4_^+^ accumulation in plants is proposed. Steady state NH_4_^+^ **control:** under 200 mM NaCl, an ideal concentration for the growth of Salicornia, GS is upregulated to detoxify cells from salinity induced NH_4_^+^ accumulation. In this phase, GS alone is able to incorporate NH_4_^+^ into glutamine and GDH is set on its glutamate deaminating mode. In this scenario, cellular NH_4_^+^ levels are maintained low. NH_4_^+^ detoxification boost: when NH_4_^+^ begins to build up (due to plants exposure to higher/toxic NaCl levels and/or an extra source of NH_4_^+^), GOGAT is deactivated due to low levels of α-ketoglutarate that is used by an intense GDH activity. The GDH activity is set on its detox mode so to incorporate NH_4_^+^ into glutamate and therefore sustain the GS ammonium detoxification function. GDH uses α-ketoglutarate to incorporate ammonium into glutamate. This will eventually lead to glutamine accumulation because GOGAT cannot convert it to glutamate. Metabolic limitations to NH_4_^+^ detoxification: in the long run (time and/or level of exposure to NH_4_^+^ and/or NaCl), glutamine accumulation will impair GS activity due to a negative feedback. Under these conditions, the only NH_4_^+^ detox enzyme is GDH, which will further deplete α-ketoglutarate and consequently lead to glutamate hyper-accumulation (since this is not anymore used by GS). High glutamate levels also lead to GDH deactivation due to a negative feedback. Consequently, NH_4_^+^ accumulation overwhelms the cellular ability to detoxify it. Under these conditions, the over-production of glutamate will be then used by P5CS to produce proline, which is a typical response in plants exposed to salt stress.

GOGAT is a key enzyme for the NH_4_^+^ assimilation pathway, linking GS and GDH activity [49] but also a general stress signaling component [50]. In contrast to GS and GDH, the gene encoding for GOGAT was downregulated when Salicornia plants were exposed to an excess of NH_4_^+^ (Figure 4M,N). GOGAT may function as modulator of the NH_4_^+^ detox machinery. The reduced activity of GOGAT leads to glutamine accumulation, which is a well-known response to NH_4_^+^ excess/toxicity in both plants and humans [51,52]. Therefore, the fast and remarkable response of GOGAT under NH_4_^+^ stress could serve as signaling intermediate [53]. Although glutamine has been shown to function as signaling molecule in response to plant nitrogen metabolism and stress response [50,54], the link between GOGAT and salt stress tolerance in plants has not been sufficiently addressed. In response to salinity, NH_4_^+^ assimilation activity could also be linked to proline accumulation [55,56,57], which is a common osmoregulator in glycophytes. Although Salicornia is likely to use glutamate (which is a precursor of proline) instead of proline as main osmoregulator [9,58], the balance between intermediate metabolites produced by an altered NH_4_^+^ assimilation pathway may play an important role in plant response to salt stress [50,54,59]. NaCl induced enzymatic activity for NH_4_^+^ incorporation into aminoacids may turn out to be an important mechanism finalized to avoid NH_4_^+^ accumulation in sensitive cellular sites [18] (Figure 6).

*S. europaea* is typical of low salt marshes, a costal ecosystem regularly flooded by saltwater or brackish water [60]. It is well known that soil nitrification is very much impaired in this environment due to the inhibition of the ammonia-oxidizing microbial community, a common phenomenon in saline and/or flooded lands [41,61,62]. This effect strongly diminishes nitrate concentration in the soil and forces plants to rely on ammonium as nitrogen source for nutrition [63]. It is likely that this environment selected plants with high ammonium assimilation rates, a functional strategy to ensure a sufficient nitrogen supply while avoiding salinity induced NH_4_^+^ toxicity [60].

## 4. Materials and Methods

### 4.1. Plant Materials and Treatments

Seeds of *S. europaea* were sterilized with 75% alcohol for 30 s and 10% sodium hypochlorite for 5 min, and finally washed 5 times using sterile ddH_2_O. Seeds of *S. europaea* were sown on agar medium plates for germination. Culture medium in plates was supplemented with ion components of ½ Hoagland nutrient mix, 1% sucrose, 1.2% agar and 0.5 g/L MES, with a pH of 6.0. 30 days old seedlings were moved to agar plates with the same basic composition of the germination plates, supplemented with two concentrations of NH_4_Cl (1 mM and 50 mM). Five concentrations of NaCl (0 mM, 1 mM, 10 mM, 50 mM, 200 mM) were combined in a factorial experimental design with 1 mM or 50 mM NH_4_Cl treatments. The plates were randomly distributed on a bench in a growth chamber. Three plates containing 6 plantlets each were sampled for biomass evaluation, ion content and enzyme activity.

### 4.2. Determination of Ion Contents

Samples were rinsed with deionized water and dried with absorbent paper, then exposed to 105 °C for 15 min fixation and subsequently oven dried at 60 °C to constant weight. The ion concentrations were determined by inductively coupled plasma emission spectrometer ICP-OES after digestion with HNO_3_-HClO_4_.

### 4.3. Hydrogen Peroxide Detection

In order to identify oxidative processes occurring in the shoots of Salicornia plantlets, Diaminobenzidine (DAB) staining method was used according to [64]. Briefly, DAB (1 mg mL^−1^) was dissolved in sterile H_2_O (pH 3.0) and maintained in the darkness. Tween 20 (25 µL) and Na_2_HPO_4_ (2.5 mL at 200 mM) were added in order to obtain a 10 mM Na_2_HPO_4_ DAB staining solution. Salicornia plantlets grown under 50 mM NH_4_Cl and 0, 1, 10, 50 and 200 mM NaCl were completely immersed in the DAB staining solution in 12-well plates. Gentle vacuum infiltration was used to ensure the complete infiltration in plants tissues. The plates were placed on a shaker (100 rpm) for 4–5 h. The solution was boiled for 15 min in bleaching solution (ethanol: acetic acid: glycerol 3:1:1) for chlorophyll degradation, allowing the visualization of brown precipitate formed by the reaction of DAB with hydrogen peroxide. After boiling, bleaching solution was replaced and pictures were taken after 30 min immersion in the fresh bleaching solution.

### 4.4. Determination of NH_4_^+^ Contents and Ammonium Assimilation Enzyme Activity

For generating a standard curve, 2 mM NH_4_Cl was dissolved using 10 mM formic acid and diluted four times by five-fold (400 μM, 80 μM, 16 μM, 3.2 μM). 10 mM formic acid was used as a blank control. 100 μL of each of the four dilutions and 900 μL OPA reaction solution (containing 3 mM o-phthalaldehyde OPA, 10 mM β-mercaptoethanol, 50 mM NaH_2_PO_3_, 50 mM Na_2_HPO_3_, pH = 6.8) were mixed and kept in a water bath at 80 °C for 15 min, then immediately cooled in an ice bath. The fluorescence absorption value was measured by using a multi-function microplate reader, and the excitation and emission wavelengths of the fluorescence detection were 410 nm and 470 nm, respectively. The standard curve was generated based on the NH_4_Cl concentration and the fluorescence absorption value.

For the detection of tissues NH_4_^+^ content, Na^+^/NH_4_^+^ treated plants were rinsed with 1 mM CaCl_2_, then the shoots and roots were separated and accurately weighed to 0.1g and collected into separate 2 mL centrifuge tube. After adding the steel balls, root or shoot samples were homogenized by a quick shock, before adding 1 mL of pre-cooled formic acid (10 mM). Samples were centrifuged at 13,000 rpm for 10 min at 4 °C. The supernatant was then transferred to a new centrifuge tube. 100 μL supernatant was mixed with 900 μL of OPA reaction solution heated at 80 °C for 15 min and immediately cooled in an ice bath. The fluorescence absorption value was measured using a Varioskan Flash with 410 nm excitation and 470 nm emission wavelengths. The NH_4_^+^ content per gram of fresh weight in shoots and roots of each sample was calculated according to the standard curve. Minor changes were made according to reported research methods [65].

The enzyme activity of glutamine synthetase (GS) and glutamate dehydrogenase (GDH) were determined using dedicated kits (Jiancheng, Nanjing, China).

### 4.5. Gene Expression Analysis Under NH_4_Cl/NaCl Treatment

For gene expression analysis, 30 day old seedlings of *S. europaea* germinated on agar were moved into a hydroponic system. The nutrient solution used was ½ Hoagland. The pH was maintained stable at 6.0 during the whole experiment. The plantlets were treated with (1) 200 mM NaCl, (2) 50 mM NH_4_Cl, (3) 200 mM NaCl + 50 mM NH_4_Cl for 0 h, 0.5 h, 2 h, 4 h, 72 h, with 0 h as control. Three plants per treatment were harvested and placed in liquid nitrogen for following gene expression analysis. Shoot and roots were collected separately, and total RNA was extracted using the RNeasy Mini Kit (Qiagen) and digested with DNase (Qiagen) to eliminate possible DNA contamination. The concentration, purity and integrity distribution of total RNA was detected by NanoDrop 2000 and 1.5% agarose gel electrophoresis. When the RNA samples had an absorbance ratio of A_260_/A_280_ = 1.9–2.1 and A_260_/A_230_ > 2.0, the samples were used for subsequent cDNA synthesis. 1 μg of total RNA was used for the synthesis of the first strand of cDNA with the reverse transcription reagent (TaKaRa). The gene CAC was used as an internal reference gene [66]. Each gene-specific primer was designed online at NCBI Blast-primer, as shown in Table S1. RT-qPCR was performed in a CFX96 Real-Time PCR Detection System (Bio-Rad) with 20 µL reaction mixture containing 10 µL of 2 × SYBR pre-mixture (BioTeke, Beijing), 4 µL of diluted cDNA (1:10), forward and reverse primers (0.25 µM), and 5 µL water. The relative gene expression was calculated according to the 2^−ΔΔCt^ method [67].

## Supplementary Materials

The following are available online at https://www.mdpi.com/2223-7747/9/2/257/s1, Table S1: List of primers used in the experiment.

## References

[1] Yarra R. (2019). The wheat NHX gene family: Potential role in improving salinity stress tolerance of plants. Plant Gene.

[2] Butcher K., Wick A.F., DeSutter T., Chatterjee A., Harmon J. (2016). Soil Salinity: A Threat to Global Food Security. Agron. J..

[3] Wang H., Wu Z., Zhou Y., Han J., Shi D. (2012). Effects of salt stress on ion balance and nitrogen metabolism in rice. Plant Soil Environ..

[4] Cheeseman J.M. (2015). The evolution of halophytes, glycophytes and crops, and its implications for food security under saline conditions. New Phytol..

[5] Ventura Y., Eshel A., Pasternak D., Sagi M. (2015). The development of halophyte-based agriculture: Past and present. Ann. Bot..

[6] Orsini F., D’Urzo M.P., Inan G., Serra S., Oh D.-H., Mickelbart M.V., Consiglio F., Li X., Jeong J.C., Yun D.-J. (2010). A comparative study of salt tolerance parameters in 11 wild relatives of *Arabidopsis thaliana*. J. Exp. Bot..

[7] Zhu J.K. (2016). Abiotic Stress Signaling and Responses in Plants. Cell.

[8] Mishra A., Tanna B. (2017). Halophytes: Potential Resources for Salt Stress Tolerance Genes and Promoters. Front. Plant Sci..

[9] Park K.W., An J.Y., Lee H.J., Son D., Sohn Y.G., Kim C.-G., Lee J.J. (2013). The growth and accumulation of osmotic solutes of the halophyte common glasswort (*Salicornia europaea*) under salinity conditions. J. Aquat. Plant Manag..

[10] Riehl T.E., Ungar I.A. (1982). Growth and ion accumulation in *Salicornia europaea* under saline field conditions. Oecologia.

[11] Lv S., Jiang P., Chen X., Fan P., Wang X., Li Y. (2012). Multiple compartmentalization of sodium conferred salt tolerance in *Salicornia europaea*. Plant Physiol. Biochem..

[12] Ma J., You J., Wang T., Xiao X., Yao Y., Zhang M., Wang J., Tian C. (2013). Global Transcriptome Profiling of *Salicornia europaea* L. Shoots under NaCl Treatment. PLoS ONE.

[13] Ventura Y., Sagi M. (2013). Halophyte crop production: The case for Salicornia and *Sarcocornia*. Environ. Exp. Bot..

[14] Momonoki Y.S., Kamimura H. (1994). Studies on the Mechanism of Salt Tolerance in *Salicornia europaea* L.: I. Changes in pH and osmotic pressure in Salicornia plants during the growth period. Jpn. J. Crop Sci..

[15] Xu C., Tang X., Shao H., Wang H. (2016). Salinity Tolerance Mechanism of Economic Halophytes From Physiological to Molecular Hierarchy for Improving Food Quality. Curr. Genom..

[16] Esteban R., Ariz I., Cruz C., Moran J.F. (2016). Review: Mechanisms of ammonium toxicity and the quest for tolerance. Plant Sci..

[17] Wei Z., Qing-jie S.U.N., Chu-fu Z., Yong-ze Y., Ji Z., Bin-bin L.U. (2004). Effect of Salt Stress on Ammonium Assimilation Enzymes of the Roots of Rice (*Oryza sativa*) Cultivars Differing in Salinity Resistance. Acta Bot. Sin..

[18] Nguyen H.T.T., Shim I.S., Kobayashi K., Usui K. (2005). Regulation of Ammonium Accumulation during Salt Stress in Rice (*Oryza sativa* L.) Seedlings. Plant Prod. Sci..

[19] Nevin J.M., Lovatt C.J. (1987). Demonstration of ammonia accumulation and toxicity in avocado leaves during water-deficit stress. Biology.

[20] Nguyen H.T.T., Shim I.S., Kobayashi K., Kenji U. (2003). Accumulation of some nitrogen compounds in response to salt stress and their relationships with salt tolerance in rice (*Oryza sativa* L.) seedlings. Plant Growth Regul..

[21] Billard J.P., Boucaud J. (1980). Effect of NaCl on the activities of glutamate synthase from a halophyte *Suaeda maritima* and from a glycophyte *Phaseolus vulgaris*. Phytochemistry.

[22] Skopelitis D.S., Paranychianakis N.V., Paschalidis K.A., Pliakonis E.D., Delis I.D., Yakoumakis D.I., Kouvarakis A., Papadakis A.K., Stephanou E.G., Roubelakis-Angelakis K.A. (2006). Abiotic Stress Generates ROS That Signal Expression of Anionic Glutamate Dehydrogenases to Form Glutamate for Proline Synthesis in Tobacco and Grapevine. Plant Cell Online.

[23] Teixeira J., Fidalgo F. (2009). Salt stress affects glutamine synthetase activity and mRNA accumulation on potato plants in an organ-dependent manner. Plant Physiol. Biochem..

[24] Meng S., Su L., Li Y., Wang Y., Zhang C., Zhao Z. (2016). Nitrate and ammonium contribute to the distinct nitrogen metabolism of *Populus simonii* during moderate salt stress. PLoS ONE.

[25] Dias A.S., De Lima G.S., Gheyi H.R., Nobre R.G., Dos Santos J.B. (2017). Emergence, Growth and Production of Sesame Under Salt Stress and Proportions of Nitrate and Ammonium. Rev. Caatinga.

[26] Fernández-Crespo E., Camañes G., García-Agustín P. (2012). Ammonium enhances resistance to salinity stress in citrus plants. J. Plant Physiol..

[27] Hessini K., Hamed K.B., Gandour M., Mejri M., Abdelly C., Cruz C. (2013). Ammonium nutrition in the halophyte *Spartina alterniflora* under salt stress: Evidence for a priming effect of ammonium?. Plant Soil.

[28] Munzarova E., Lorenzen B., Brix H., Vojtiskova L., Votrubova O. (2006). Effect of NH_4_^+^/NO_3_^−^ availability on nitrate reductase activity and nitrogen accumulation in wetland helophytes *Phragmites australis* and *Glyceria maxima*. Environ. Exp. Bot..

[29] Camañes G., Cerezo M., Primo-Millo E., Gojon A., García-Agustín P. (2009). Ammonium transport and CitAMT1 expression are regulated by N in Citrus plants. Planta.

[30] Ma J., Xiao X., Li L., Maggio A., Zhang D., Abdelshafy Mohamad O.A., Van Oosten M., Huang G., Sun Y., Tian C. (2018). Large-scale de novo transcriptome analysis reveals specific gene expression and novel simple sequence repeats markers in salinized roots of the euhalophyte *Salicornia europaea*. Acta Physiol. Plant..

[31] Pang Q., Chen S., Dai S., Chen Y., Wang Y., Yan X. (2010). Comparative proteomics of salt tolerance in arabidopsis thaliana and thellungiella halophila. J. Proteome Res..

[32] Kosová K., Prášil I.T., Vítámvás P. (2013). Protein contribution to plant salinity response and tolerance acquisition. Int. J. Mol. Sci..

[33] Wang X., Fan P., Song H., Chen X., Li X., Li Y. (2009). Comparative Proteomic Analysis of Differentially Expressed Proteins in Shoots of *Salicornia europaea* under Different Salinity. J. Proteome Res..

[34] Razzaghi Komaresofla B., Alikhani H.A., Etesami H., Khoshkholgh-Sima N.A. (2019). Improved growth and salinity tolerance of the halophyte *Salicornia* sp. by co–inoculation with endophytic and rhizosphere bacteria. Appl. Soil Ecol..

[35] Benjamin J.J., Lucini L., Jothiramshekar S., Parida A. (2019). Metabolomic insights into the mechanisms underlying tolerance to salinity in different halophytes. Plant Physiol. Biochem..

[36] Jahn T.P., Schjoerring J.K., Cuin T.A., Pedas P., Shabala S., Hegelund J.N., Hoopen F.T. (2010). Competition between uptake of ammonium and potassium in barley and Arabidopsis roots: Molecular mechanisms and physiological consequences. J. Exp. Bot..

[37] Orsini F., Alnayef M., Bona S., Maggio A., Gianquinto G. (2012). Low stomatal density and reduced transpiration facilitate strawberry adaptation to salinity. Environ. Exp. Bot..

[38] Almeida D.M., Margarida Oliveira M., Saibo N.J.M. (2017). Regulation of Na^+^ and K^+^ homeostasis in plants: Towards improved salt stress tolerance in crop plants. Genet. Mol. Biol..

[39] Frechilla S., Lasa B., Ibarretxe L., Lamsfus C., Aparicio-Tejo P. (2001). Pea responses to saline stress is affected by the source of nitrogen nutrition (ammonium or nitrate). Plant Growth Regul..

[40] Katschnig D., Bliek T., Rozema J., Schat H. (2015). Constitutive high-level *SOS1* expression and absence of *HKT1;1* expression in the salt-accumulating halophyte Salicornia *dolichostachya*. Plant Sci..

[41] Britto D.T., Kronzucker H.J. (2002). NH_4_^+^ toxicity in higher plants: A critical review I. Introduction. J. Plant Physiol..

[42] Coleto I., Vega-Mas I., Glauser G., González-Moro M.B., Marino D., Ariz I. (2019). New insights on *Arabidopsis thaliana* root adaption to ammonium nutrition by the use of a quantitative proteomic approach. Int. J. Mol. Sci..

[43] Jian S., Liao Q., Song H., Liu Q., Lepo J.E., Guan C., Zhang J., Ismail A.M., Zhang Z. (2018). NRT1.1-Related NH_4_^+^ Toxicity Is Associated with a Disturbed Balance between NH_4_^+^ Uptake and Assimilation. Plant Physiol..

[44] O’Neal D., Joy K.W. (1974). Glutamine Synthetase of Pea Leaves. Plant Physiol..

[45] Bottacin A., Cacco G., Saccomani M. (1985). Nitrogen absorption and assimilation in NaCl-resistant and NaCl-susceptible millet genotypes (*Pennisetum americanum*). Can. J. Bot..

[46] Abouelsaad I., Weihrauch D., Renault S. (2016). Effects of salt stress on the expression of key genes related to nitrogen assimilation and transport in the roots of the cultivated tomato and its wild salt-tolerant relative. Sci. Hortic. (Amsterdam).

[47] Cruz C., Bio A.F.M., Domínguez-Valdivia M.D., Aparicio-Tejo P.M., Lamsfus C., Martins-Loução M.A. (2006). How does glutamine synthetase activity determine plant tolerance to ammonium?. Planta.

[48] Fontaine J.-X., Tercé-Laforgue T., Armengaud P., Clément G., Renou J.-P., Pelletier S., Catterou M., Azzopardi M., Gibon Y., Lea P.J. (2012). Characterization of a NADH-Dependent Glutamate Dehydrogenase Mutant of Arabidopsis Demonstrates the Key Role of this Enzyme in Root Carbon and Nitrogen Metabolism. Plant Cell.

[49] Konishi N., Ishiyama K., Matsuoka K., Maru I., Hayakawa T., Yamaya T., Kojima S. (2014). NADH-dependent glutamate synthase plays a crucial role in assimilating ammonium in the Arabidopsis root. Physiol. Plant..

[50] Kan C.C., Chung T.Y., Juo Y.A., Hsieh M.H. (2015). Glutamine rapidly induces the expression of key transcription factor genes involved in nitrogen and stress responses in rice roots. BMC Genom..

[51] Rigano C., Di Martino Rigano V., Vona V., Carfagna S., Carillo P., Esposito S. (1996). Ammonium assimilation by young plants of *Hordeum vulgare* in light and darkness: Effects on respiratory oxygen consumption by roots. New Phytol..

[52] Albrecht J., Norenberg M.D. (2006). Glutamine: A Trojan horse in ammonia neurotoxicity. Hepatology.

[53] Egea I., Albaladejo I., Meco V., Morales B., Sevilla A., Bolarin M.C., Flores F.B. (2018). The drought-tolerant *Solanum pennellii* regulates leaf water loss and induces genes involved in amino acid and ethylene/jasmonate metabolism under dehydration. Sci. Rep..

[54] Ferrario-Mery S., Masclaux C., Suzuki A., Valadier M.H., Hirel B., Foyer C.H. (2001). Glutamine and α-ketoglutarate are metabolite signals involved in nitrate reductase gene transcription in untransformed and transformed tobacco plants deficient in ferredoxinglutamine-α-ketoglutarate aminotransferase. Planta.

[55] Viégas R.A., Albenísio J. (1999). Ammonia assimilation and proline accumulation in young cashew plants during long term exposure to NaCl-salinity. Rev. Bras. Fisiol. Veg..

[56] Wang Z.Q., Yuan Y.Z., Ou J.Q., Lin Q.H., Zhang C.F. (2007). Glutamine synthetase and glutamate dehydrogenase contribute differentially to proline accumulation in leaves of wheat (*Triticum aestivum*) seedlings exposed to different salinity. J. Plant Physiol..

[57] Lee B.R., Muneer S., Park S.H., Zhang Q., Kim T.H. (2013). Ammonium-induced proline and sucrose accumulation, and their significance in antioxidative activity and osmotic adjustment. Acta Physiol. Plant..

[58] Kovaleva N.P., Ushakova S.A., Tikhomirova N.A., Kolmakova A.A., Gribovskaya I.V. (2006). Effect of photosynthetically active radiation, salinization, and type of nitrogen nutrition on growth of *Salicornia europaea* plants. Russ. J. Plant Physiol..

[59] Forde B.G., Lea P.J. (2007). Glutamate in plants: Metabolism, regulation, and signalling. J. Exp. Bot..

[60] Egan T.P., Ungar I.A. (2000). Mortality of the Salt Marsh Species *Salicornia europaea* and *Atriplex Prostrata* (Chenopodiaceae ) in Response to Inundation. Ohio J. Sci..

[61] Cui Y.W., Zhang H.Y., Ding J.R., Peng Y.Z. (2016). The effects of salinity on nitrification using halophilic nitrifiers in a Sequencing Batch Reactor treating hypersaline wastewater. Sci. Rep..

[62] Tissue D.T., Nguyen L.T.T., Bange M.P., Anderson I.C., Singh B.K., Braunack M., Osanai Y. (2018). Impacts of waterlogging on soil nitrification and ammonia-oxidizing communities in farming system. Plant Soil.

[63] Tho B.T., Lambertini C., Eller F., Brix H., Sorrell B.K. (2017). Ammonium and nitrate are both suitable inorganic nitrogen forms for the highly productive wetland grass *Arundo donax*, a candidate species for wetland paludiculture. Ecol. Eng..

[64] Daudi A. (2016). Detection of Hydrogen Peroxide by DAB Staining in Arabidopsis Leaves. Bio. Protoc..

[65] Husted S., Hebbern C., Mattsson M., Schjoerring J. (2001). A critical experimental evaluation of methods for determination of NH_4_^+^ in plant tissue, xylem sap and apoplastic fluid. Physiol. Plant..

[66] Xiao X., Ma J., Wang J., Wu X., Li P., Yao Y. (2015). Validation of suitable reference genes for gene expression analysis in the halophyte *Salicornia europaea* by real-time quantitative PCR. Front. Plant Sci..

[67] Livak K.J., Schmittgen T.D. (2001). Analysis of Relative Gene Expression Data Using Real-Time Quantitative PCR and the 2^−ΔΔCT^ Method. Methods.

