# Supplementary material for: Regulation of Ammonium Cellular Levels is An Important Adaptive Trait for the Euhalophytic Behavior of Salicornia europaea
Source: Plants (Basel). 2020 Feb 17;9(2):257. doi: 10.3390/plants9020257 (PMC7076498; doi:10.3390/plants9020257)
Supplement: Supplementary file 1 [file plants-09-00257-s001.pdf]

Table S1. List of primers used in the experiment.

Unigene	Nr annotation	Gene	Primer pairs：F/R	Size (bp)	Efficiency
Unigene5393_All	cytosolic glutamine synthetase	GS1	TGATCCAAAGCCCATCCCTG/ TGTTAGCCACTCCCCACAAG	230	1.975
Unigene29790_All	plastid glutamine synthetase 2	GS2	TGATGGTTCGAGCACAGGAC/ TCTCAGCAGCTTTGTGTCGT	173	2.074
Unigene54171_All	NADH-dependent glutamate synthase 1	GOGAT1	GGGTGAACCTCATGGCTGAA/ CCTGACAAGTCCCGTCCTTC	169	2.185
Unigene70174_All	Ferredoxin-dependent glutamate synthase	GOAGT2	ATTGGTCCCTTTGATCGGCT/ GGGCATGGTGTCTTTTGCATT	189	1.937
Unigene5295_All	NADH-glutamate dehydrogenase	GDH	GCATAGGCTGTAACCCGTCT/ TCAGGAGCTGGTACGTCAGT	115	1.918
Unigene31433_All	Clathrin adaptor complexes	CAC	CGTGCCTTCTGATGCGACTA/ TGCCTCTTCACTTGTGATGCT	166	2.028
